# Divergence of Genes Encoding *CITED1* and *CITED2* in Blunt Snout Bream (*Megalobrama amblycephala*) and Their Transcriptional Responses to Hypoxia

**DOI:** 10.3389/fphys.2018.00186

**Published:** 2018-03-06

**Authors:** Yuan Sun, Hong-Hong Guo, Dan-Dan Guo, Xia-Yun Jiang, Shu-Ming Zou

**Affiliations:** Key Laboratory of Freshwater Aquatic Genetic Resources, Ministry of Agriculture, Genetics and Breeding Center for Blunt Snout Bream, Shanghai Ocean University, Shanghai, China

**Keywords:** blunt snout bream, *CITED1*, *CITED2*, hypoxia, transcriptional responses

## Abstract

The proteins CITED belong to a family of non-DNA-binding transcriptional co-regulators involved in the regulation of various transcriptional responses. Previous studies suggest that members of CITED family may function in response to hypoxia in mammals. however, the molecular and functional information on *CITED* genes in aquaculture fish is unclear. Here, we characterized and examined the transcriptional patterns of *CITED1* and *CITED2* genes in the hypoxia-sensitive blunt snout bream (*Megalobrama amblycephala*). Blunt snout bream *CITED1* and *CITED2* genes shared a relatively low sequence identity of 45%. *CITED1* and *CITED2* mRNAs were widely transcribed in adult tissues. During embryogenesis, *CITED1* mRNA was significantly transcribed at 4, 24, 28, 40, and 44 hpf, whereas *CITED2* mRNA levels fluctuated from the zygote to 44 hpf larval stage. Whole-mount *in situ* hybridization demonstrated that *CITED1* and *CITED2* mRNAs were detected in the brain at 12 hpf, brain and gut at 24 hpf, and brain at 36 hpf. In addition, low expression of *CITED1* mRNA was detected in the tailbud at 24 hpf. The results of acute hypoxia experiment showed that *CITED1* and *CITED2* mRNAs were markedly upregulated in the kidney and downregulated in the liver, brain, gill, and heart under hypoxia. Embryos in hypoxic conditions at different developmental stages showed a significant increase in mRNA levels of *CITED1* and *CITED2*. These results provide a new insight into the divergence of *CITED1* and *CITED2* genes and their transcriptional responses to hypoxia.

## Introduction

The proteins CITED, cAMP-responsive element-binding protein (CBP)/p300-interacting transactivator with glutamic acid (E) and aspartic acid (D)-rich C-terminal domain, are involved in the modulation of a variety of cellular and developmental processes and responses to environmental stimuli at the transcriptional level in mammals (Dunwoodie et al., 1998; Yahata et al., 2001). To date, four different homologs (*CITED1-4*) have been reported in vertebrates (Bhattacharya et al., 1999). *CITED1*, formally known as *MSG1*, was first isolated in 1996 from murine melanoma B16-F1 cells (Shioda et al., 1996) and mapped to chromosome Xq13.1 in humans (Fenner et al., 1998). *CITED2*, originally called as *MRG1* (*MSG1*-related gene 1) shares transcription-activating CR1 (14 amino acid residues) and CR2 (~50 amino acid residues) domains with *CITED1* (Shioda et al., 1997). Hypoxic conditions lead to the formation of a DNA-binding complex containing both hypoxia-inducible factor 1 (HIF-1) alpha and CBP/p300. Hence, CBP/p300-HIF complexes participate in the induction of hypoxia-responsive genes (Bracken et al., 2003; Kinoshita et al., 2004; Shen et al., 2010). Members of CITED family may bind to CBP/p300 with a strong affinity and negatively regulate HIF-1 transactivation (Bhattacharya et al., 1999; Ng et al., 2010).

Although CITED family plays diverse roles in mammals (Dunwoodie et al., 1998; Schlange et al., 2000; Bamforth et al., 2001; Yahata et al., 2001, 2002), their functions related to oxygen regulation are questionable. Recent studies in teleost fish have highlighted their transcriptional activities responsible to alleviate the effect of hypoxia and fine-tune a number of signaling pathways coping with oxygen change (Ng et al., 2003, 2009, 2010; van den Beucken et al., 2007; Yoon et al., 2011). To date, CITED genes have been identified in zebrafish and grass carp genome (Ng et al., 2003, 2009, 2010; Thisse et al., 2004). *CITED* genes in grass carp may be transcribed in a negative feedback loop to regulate HIF-1 transactivation in response to hypoxia stress (Ng et al., 2009, 2010). Although the information on the embryonic expression of *CITED1* in zebrafish is lacking, *CITED2* mRNAs are transcribed in the brain and a part of cephalic nervous system using whole-mount *in situ* hybridization (Thisse et al., 2004). In addition, our previous transcriptome results of blunt snout bream have shown that both *CITED1* and *CITED2* genes are associated with the expression of hypoxia by transcriptome analysis (Li et al., 2015). Thus, further studies are essential to determine the transcriptional responses of *CITED1* and *CITED2* at the molecular level in important aquaculture fish species.

As a principal herbivorous species in freshwater fish polyculture systems, blunt snout bream (*Megalobrama amblycephala*) is widely favored as a delicacy in China. The production output of blunt snout bream was more than 0.7 million tons in 2015 (FBMA., 2016). Temperature fluctuations, low photosynthetic activity, and low or stagnant water flow may cause hypoxia in fish culture ponds (Karim et al., 2003; Martínez et al., 2011). A short period (<2 h) of hypoxia (less than 0.5 mg/L) at room temperature may be lethal (Shen et al., 2010; Tian et al., 2014; Li et al., 2015). Blunt snout bream, an extremely hypoxia-sensitive fish, has a relatively high critical oxygen tension of 0.9 mg/L at 10°C, at which it loses its equilibrium (LOE_crit_) (Wu et al., 2017). Based on the differential expression patterns of *CITED1* and *CITED12* genes during transcriptome analysis of blunt snout bream under hypoxia condition (GenBank SRP050593) (Li et al., 2015), we analyzed the molecular and spatiotemporal expression patterns as well as the transcriptional responses of these genes to acute hypoxia.

## Materials and methods

### Experimental fish culture and treatment

All experiments were conducted following the guidelines approved by the Shanghai Ocean University Committee on the Use and Care of Animals. Blunt snout bream was obtained from the Bream Genetics and Breeding Center of Shanghai Ocean University, Shanghai, China. Fertilized eggs were generated by artificial insemination. Fertilized eggs (100–200) were plated in each Petri dish (15 cm in diameter). Egg fertilization and embryo development was carried out at room temperature (~20°C). During embryogenesis, water in the Petri dish was replaced every 4 h with well-aerated water to maintain the normal value of dissolved oxygen (DO) of 7.0 ± 0.5 mg/L (Ouyang et al., 2001). DO was monitored continuously using a dissolved oxygen meter (YSI Pro-ODO, Germany). Embryos were stored every 4 h after fertilization (0 ~ 44 hpf) by immersing in RNA Store (Tiangen, Shanghai, China) and kept in 4°C overnight and then −80°C until used. Five adult blunt snout bream were euthanized by immersion in MS-222 (Sigma, St. Louis, MO, USA). The weight of 2-year-old adult blunt snout bream was 1,000 g according to the experimental requirements. Tissues (brain, gill, eyes, muscle, skin, heart, liver, spleen, kidney, intestine, and gonad) were rapidly dissected, frozen in liquid nitrogen and stored at −80°C until use.

### Hypoxia treatments of embryos

Embryos were subjected to hypoxia as recently reported (Zhang et al., 2017). During embryogenesis, water in the Petri dish was replaced with well-aerated slow-flowing water to maintain the normal value of dissolved oxygen (DO) of 7.0 ± 0.5 mg/L at room temperature (~20°C). Blunt snout bream embryos at different developmental stages (2, 8, 14, 20, and 26 hpf) were exposed to severe hypoxic DO conditions (3.5 ± 0.5 mg/L) for 6 h (see Zhang et al., 2017). Oxygen was depleted by bubbling water with nitrogen gas (Zhang et al., 2003). The embryos at similar stages under normoxic DO conditions were used as controls. After each exposure period, 20 embryos were submersed in RNA store (Tiangen, Shanghai, China), maintained at 4°C overnight, and stored at −80°C until total RNA isolation. Each experiment was performed in triplicates.

### Hypoxia treatments on juvenile fish

For the juvenile fish treatment, 30 juvenile blunt snout bream (150~160 g each) were randomly transferred into three 50 L tanks within a continuous flow system (10 individual per tank). After 1 week of acclimation, two groups were exposed to severe hypoxic DO conditions (DO = 1.0 ± 0.5 mg/L) for 4 h by nitrogen-filled manipulation and a control group was retained under normoxic DO conditions (DO = 7.0 ± 0.5 mg/L) (Guan et al., 2017). Another two parallels were set for the control and experimental groups, respectively. After the exposure period, five fish from each experimental group and the control group were sacrificed. Then DO levels of two hypoxic treatment groups were adjusted to normal levels within 1 h by bubbling air. The remaining five fish from each recovery treatment and the control group were sampled after 24 h. Tissues were immediately excised, frozen in liquid nitrogen and stored at −80°C until use.

### Molecular cloning of blunt snout bream *CITED1* and *CITED2* cDNAs

Total RNA was isolated from blunt snout bream embryos at 44 hpf using TRIzol reagent (Invitrogen, Carlsbad, CA,USA) and subsequently treated with DNase (Promega, Madison, WI, USA) to remove contaminant genomic DNA. First-strand cDNA was reverse transcribed from the total RNA by using reverse transcriptase M-MLV (TaKaRa, Japan) with oligo-dT primers according to the manufacture's instructions. The primer pairs for *CITED1* and *CITED2* mRNA were designed based on transcriptome data of blunt snout bream (Li et al., 2015). PCR was performed to amplify partial cDNA fragments of blunt snout bream *CITED1* by using the primers *CITED1*-F and *CITED1*-R (Table 1). A 315-bp partial PCR fragment of blunt snout bream *CITED1* was cloned, sequenced, and used to design nested gene-specific primers for 3′rapid amplification of cDNA ends (RACE) analysis and 5′RACE analysis (Table 1). And similar to the above method, a 460-bp partial PCR fragment of blunt snout bream *CITED2* was cloned, sequenced, and used to design nested gene-specific primers for 3′RACE analysis and 5′RACE analysis (Table 1). The 5′ and 3′ ends of *CITED1 and CITED2* mRNAs were amplified using the SMART RACE cDNA amplification kit (Clontech, Mountain View, CA, USA). PCR products were gel-purified, ligated into the cloning vector pGEM-T (Promega, Madison, WI, USA), and transformed into *Escherichia coli DH5*α competent cells. Positive clones were examined by PCR and direct sequencing.

**Table 1:** Primer sequences used in this study.

**Primers name**	**Primer sequence (5′-3′)**
CITED1-RT-F1	CACTCTCCCTCCTGCACTAC
CITED1-RT-R1	GCGACATCAGAACTTCCTCATC
CITED2-RT-F1	CCGCAGCAGCAGCATTAT
CITED2-RT-R1	CCAAGCCCATTTCTATCA
CITED1-3RACE-O	CGGCACAGACACCTGGGAT
CITED1-3RACE-I	GATGAGGAAGTTCTGATGTCGC
CITED1-5RACE-O	GCAGCAGGACTGGAAACA
CITED1-5RACE-I	AGGGCTCGTCTTGCCTGAAC
CITED2-3RACE-O	GTCTGGGGTTCCTCTGCCTGG
CITED2-3RACE-I	TTATTGACGAGGAGGTCTTGATG
CITED2-5RACE-O	CTGGGATTGTCAGTTACTTTTTGCT
CITED2-5RACE-I	GTGATTTGCGTTTACATTCCCTCCC
CITED1-in situ-F	CCAGCACCTCATAGCCTCC
CITED1-in situ-R	ACGGCTTCTCAAGGGAATCTCA
CITED2-in situ-F	TTGACGAGGAGGTCTTGATGT
CITED2-in situ-R	TGTTCTGTGGGTTTATCGTTTG
CITED1-q RT-F	CGACTTCGGATCTGTTCTTG
CITED1-q RT-R	GTGCAGGAGGGAGAGAGAGT
CITED2-q RT-F	AGACGCGTGTGTTTATTCGT
CITED2-q RT-R	AATGTTGTTGCTGTGGAAGC
18S-q RT-F	ACCGCAGCTAGGAATAATGG
18S-q RT-R	GGTCGGAACTACGACGGTAT

### Sequence and phylogenetic analyzes

The putative sequences of *CITED1* and *CITED2* protein from different species were compared using the National Center for Biotechnology Information BLASTP search program. Nucleotide sequences of *CITED1* and *CITED2* were analyzed using BioEdit 7.0.0.1 (Jeon et al., 2014). Alignment of putative amino acid sequences of the *CITED1* and *CITED2* proteins was performed with Clustal X 1.81 (Thompson et al., 1997). Phylogenetic analysis was performed using coding sequences with the neighbor-joining (NJ) method in MEGA 6.06 (Tamura et al., 2013). Gap sites in the alignment were used for the phylogenetic reconstruction. Reliability of the estimated tree was evaluated using the bootstrap method with 1,000 pseudo-replications.

### Whole-mount *in situ* hybridization

A 853-bp PCR fragment of blunt snout bream *CITED1* amplified by primers *CITED1*-In situ-F/-R (Table 1) and a 509-bp PCR fragment of *CITED2* amplified by primers *CITED2*-In situ-F/-R (Table 1) were subcloned into the pGEM-T vector (Tiangen, Beijing, China). Each template plasmids was linearized by restriction enzyme digestion, followed by *in vitro* transcription with SP6 or T7 RNA polymerase to generate the antisense or sense RNA riboprobes, respectively. Fixed embryos were washed briefly in PBS containing 0.1% Tween-20, transferred to 100% methanol, and stored at −20°C for a minimum of 24 h. Whole-mount *in situ* hybridization using digoxigenin (DIG)-labeled RNA riboprobes was performed as reported previously (Jiang et al., 2012). Embryos were hybridized with appropriate riboprobes at 60°C, incubated with anti-DIG antibodies conjugated with alkaline phosphatase (AP) and stained with Roche BM Purple AP substrates (Roche, Basel, Switzerland) to produce purple, insoluble precipitates. Embryos were photographed using a Nikon SMZ1500 fluorescence microscope (Tokyo, Japan).

### Quantitative real-time PCR

Total RNAs were isolated from embryos at the same developmental stage during embryogenesis or from tissues of blunt snout bream by using the TRIzol reagent (Invitrogen). After DNase treatment, 600 ng total RNA was reverse-transcribed to single-strand cDNA using a Prime Script RT reagent kit (TaKaRa, Japan) according to the manufacturer's instructions. Quantitative real-time PCR (qPCR) was carried out on a CFX96 Touch™ real-time PCR Detection System (BioRad, Hercules, CA, USA) according to the manufacturer′s instructions. The hypoxia stable 18S gene was amplified by 18S-qRT-F/-R (Table 1) and served as the internal reference. Primer pairs were *CITED1*-qRT-F/-R and *CITED2*-qRT-F/-R (Table 1). The qPCR was performed using SYBR Green Premix Ex Taq (TaKaRa, Japan). The program for qRT-PCR was 95°C for 30 s and 40 cycles of amplification at 95°C for 5 s, 59.5°C for 20 s, and 72°C for 15 s. The molecular number of a particular gene transcript was calculated based on the standard curve and normalized to the 18S mRNA level. Each experiment was repeated in triplicate. The expression level of different genes was analyzed using the comparative CT method (2^−ΔΔ*CT*^ method) (Livak and Schmittgen, 2001) after the PCR program.

### Statistical analysis

Data from the qRT-PCR are expressed as means ± SE. Differences among groups were analyzed with a one-way ANOVA followed by Fisher′ s *post-hoc* tests or unpaired *t*-tests. Significance was accepted at the level of *p* < 0.01.

## Results

### Identification of *CITED1* and *CITED2* cDNAs in blunt snout bream

**Figure 1:**
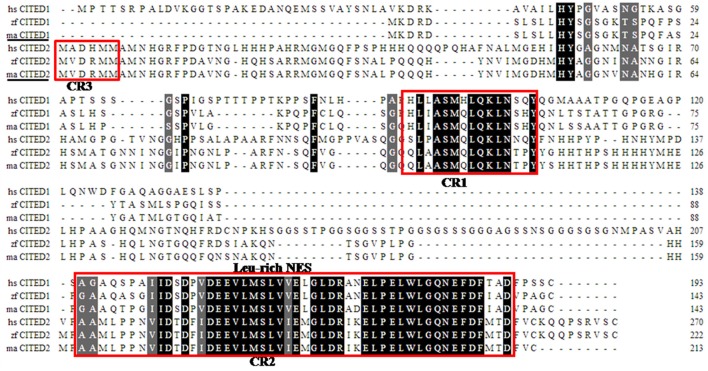
Alignment of deduced blunt snout bream (ma) CITED1 and−2 amino acid sequences with zebrafish (zf) and human (hs) homologs. The CITED domain (CR1, CR2, CR3) is indicated by red boxes, and the CITED signature (Leu-rich NES) is indicated by red frame.

The full-length cDNA sequences of both *CITED1* (GenBank Acc. No. KX913945) and *CITED2* (GenBank Acc. No. KX913946) genes were obtained in blunt snout bream. *CITED1* cDNA was 1,459 bp in length and comprised a 249 bp 5′-untranslated region (5′-UTR), a 432 bp open reading frame (ORF) encoding 143 amino acid residues and a 778 bp 3′-UTR including a poly-(A) tail (Figure 1). The *CITED2* cDNA was 1,530 bp in length, containing a 291 bp 5′-UTR, 642 bp ORF encoding 213 amino acid residues and 598 bp 3′-UTR.

**Figure 2:**
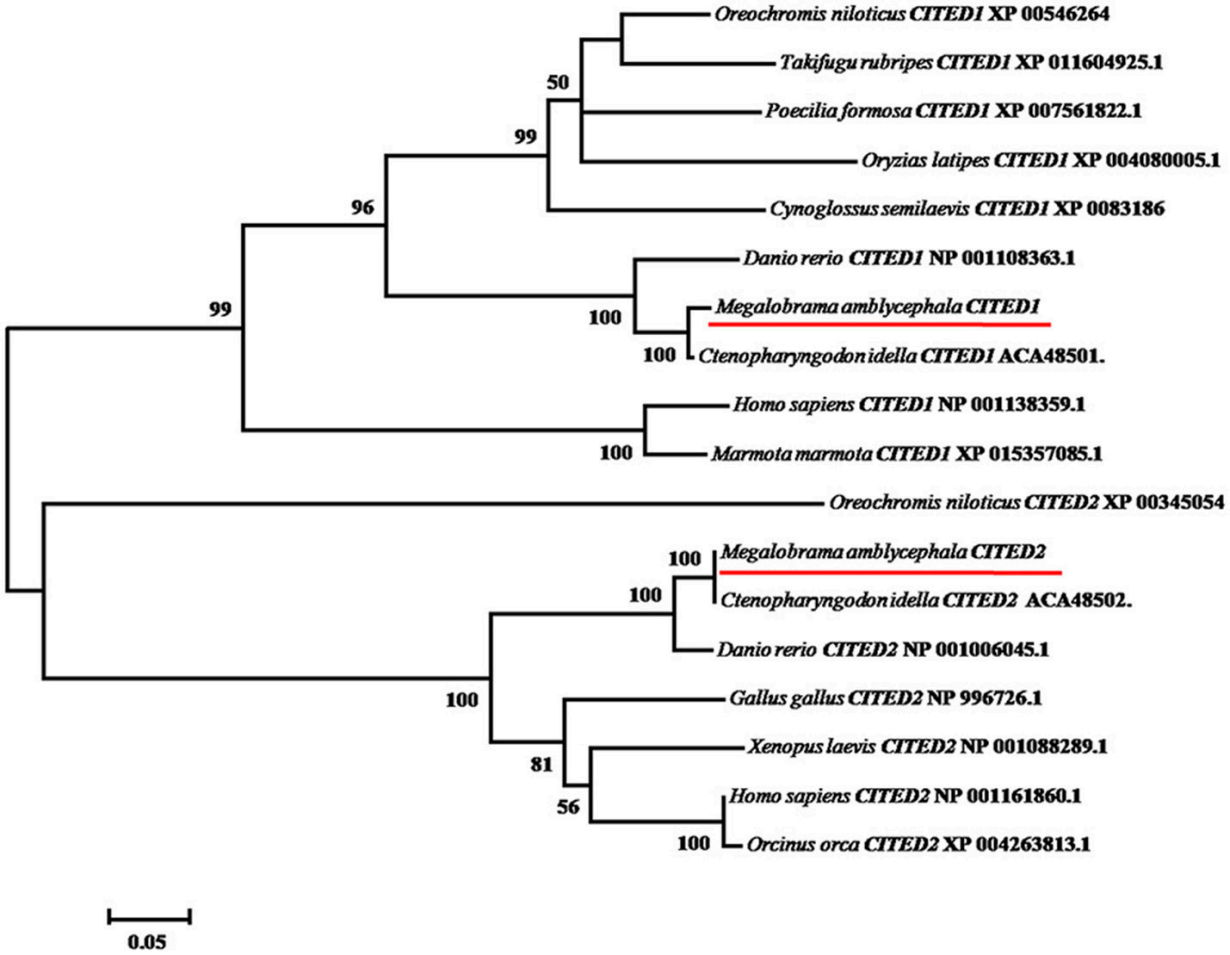
Phylogenetic analysis of vertebrate CITED1 and−2. Accession numbers of sequences retrieved from GenBank and European Molecular Biology Laboratory are shown. The tree was constructed by the neighbor-joining method using MEGA 6.06 software. The bootstrap values derived from 1,000 replications are shown. Objective gene has been highlighted in red line.

Multiple alignments suggest that both CITED1 and CITED2 peptides from blunt snout bream were predicted to consist of CR1 and CR2 domains displaying a leucine-rich nuclear-export signal (Leu-rich NES), as observed in other species. (Figure 1). In addition, CITED2 peptide displayed CR3 domain (Figure 1). Pairwise comparison showed that the mature peptides CITED1 and CITED2 had low sequence identities (45%). Sequence analysis showed that CITED1 protein of blunt snout bream shared high sequence identity with CITED1 from grass carp (94%) and zebrafish (83%). Moreover, CITED2 protein of blunt snout bream shared high sequence identity with CITED2 from grass carp (95%) and zebrafish (93%). The phylogenetic tree demonstrated that *CITED1* and *CITED2* of blunt snout bream are well clustered with their orthologs in other species (Figure 2).

### Transcriptional patterns in embryo and adult tissues of *CITED1* and *CITED2* mRNAs

**Figure 3:**
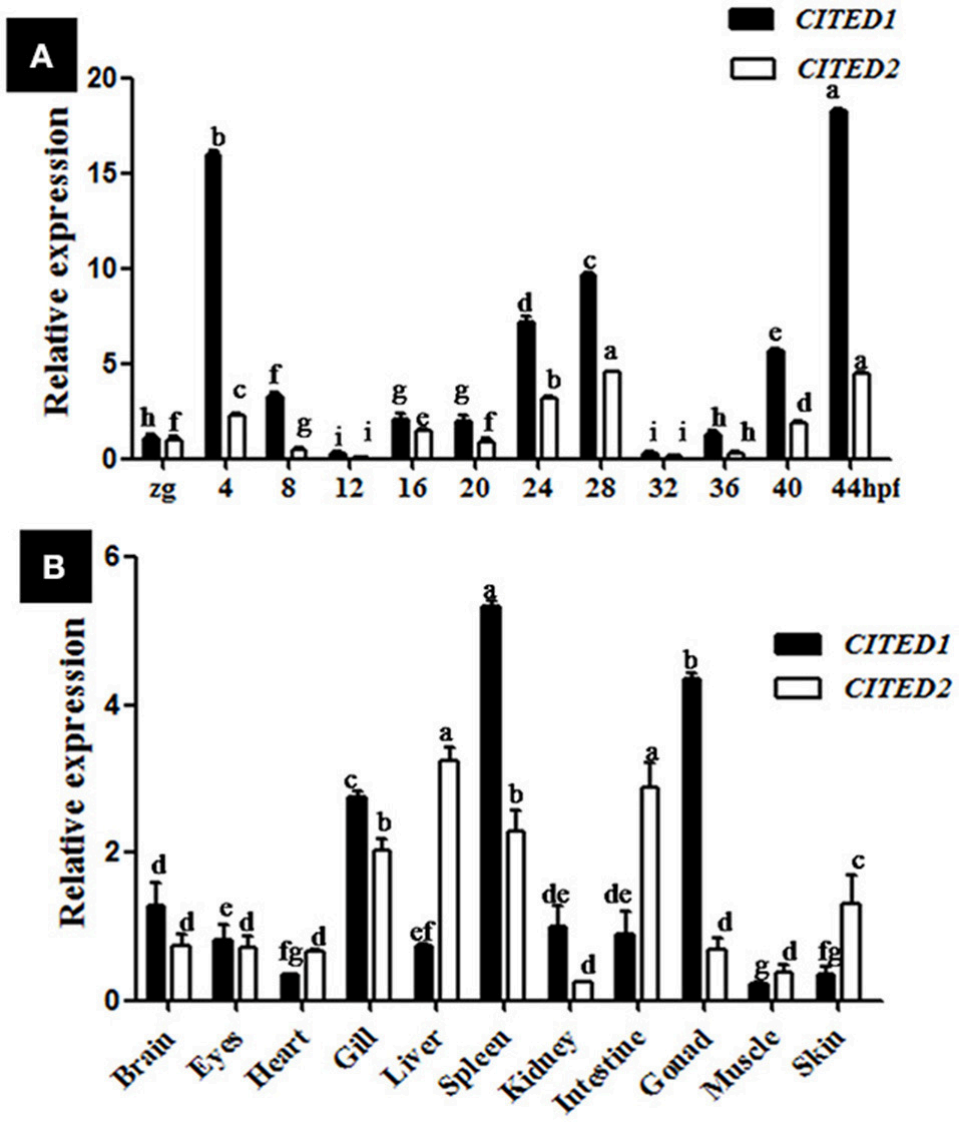
Expression of *CITED1*/*-2* mRNAs during embryogenesis **(A)** and in adult tissues **(B)** of blunt snout bream. The relative expression was analyzed by *q*RT-PCR. The *CITED1*/*-2* copy numbers were normalized to the amount of 18S mRNA. The results are given as mean ± SE for separate samples (*n* = 5). Columns marked with different letters are significantly different (*p* < 0.01). Zg, zygote; hpf, hours post-fertilization.

**Figure 4:**
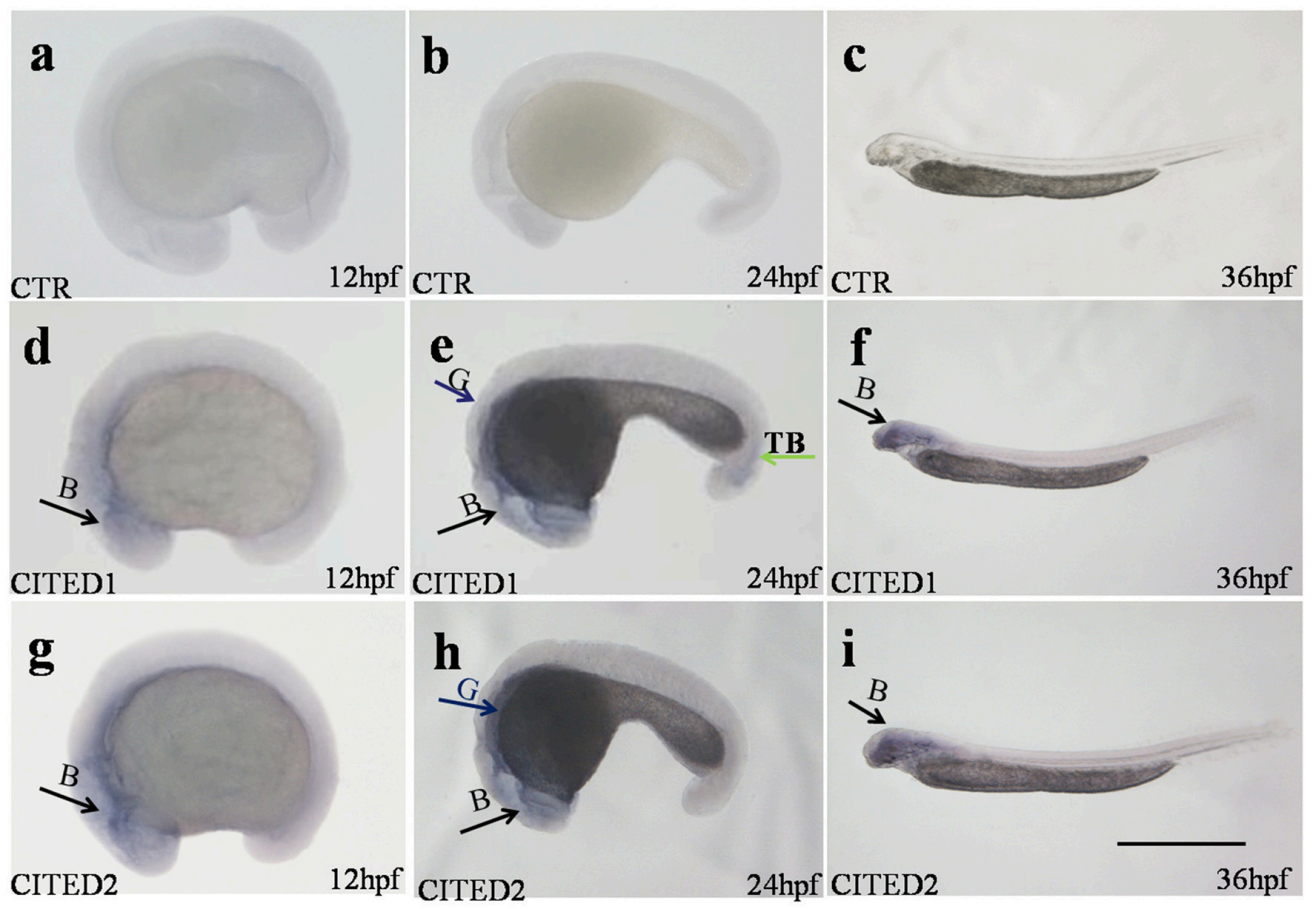
Whole-mount embryo *in situ* hybridization analysis at different embryonic stages of *CITED1* /*-2* mRNA of blunt snout bream. *CITED1* antisense probe **(D–F)**, *CITED2* antisense probe **(G–I)**, and *CITED1* or *-2* sense probe **(A–C)** were used. All embryos are viewed laterally with the head to the left. B, brain; G, gut; TB, tail bud. Scale bar = 500 μm.

Both *CITED1* and *CITED2* mRNAs were maternally deposited, as their transcripts were detected in the zygote. Blunt snout bream *CITED1* mRNA was significantly expressed (*p* < 0.01) at 4, 24, 28, 40, and 44 hpf (Figure 3A). On the other hand, *CITED2* mRNA fluctuated from the zygote to 44 hpf larvae (Figure 3A). Both *CITED1* and *CITED2* mRNAs were detected in multiple adult tissues in blunt snout bream. *CITED1* mRNA was highly expressed in the spleen, gonad, and gill and moderately expressed in the brain, kidney, intestine, eyes, and liver. The expression of *CITED1* mRNA was relatively low in the muscle, heart, and skin (Figure 3B). *CITED2* mRNA was highly expressed in the liver, intestine, spleen, and gill, while its expression was moderate in the skin, brain, eyes, heart, and gonad. Kidney and muscle tissues showed relatively low level of *CITED2* mRNA expression (Figure 3B). *CITED1* or *CITED2* sense probe (Figures 4A–C), *CITED1* antisense probe (Figures 4D–F) and *CITED2* antisense probe (Figures 4G–I) were detected in the whole-mount embryo during the *in situ* hybridization analysis of embryos at different developmental stages. Whole-mount *in situ* hybridization results showed that both *CITED1* and *CITED2* mRNAs were detected in the brain at 12 hpf (Figures 4D,G), brain and gut at 24 hpf (Figures 4E,H), and brain at 36 hpf (Figures 4F,I). In addition, weak expression of *CITED1* mRNA was detected in the tailbud at 24 hpf (Figure 4E).

### Transcriptional responses of *CITED1* and *CITED2* to hypoxia during embryogenesis

**Figure 5:**
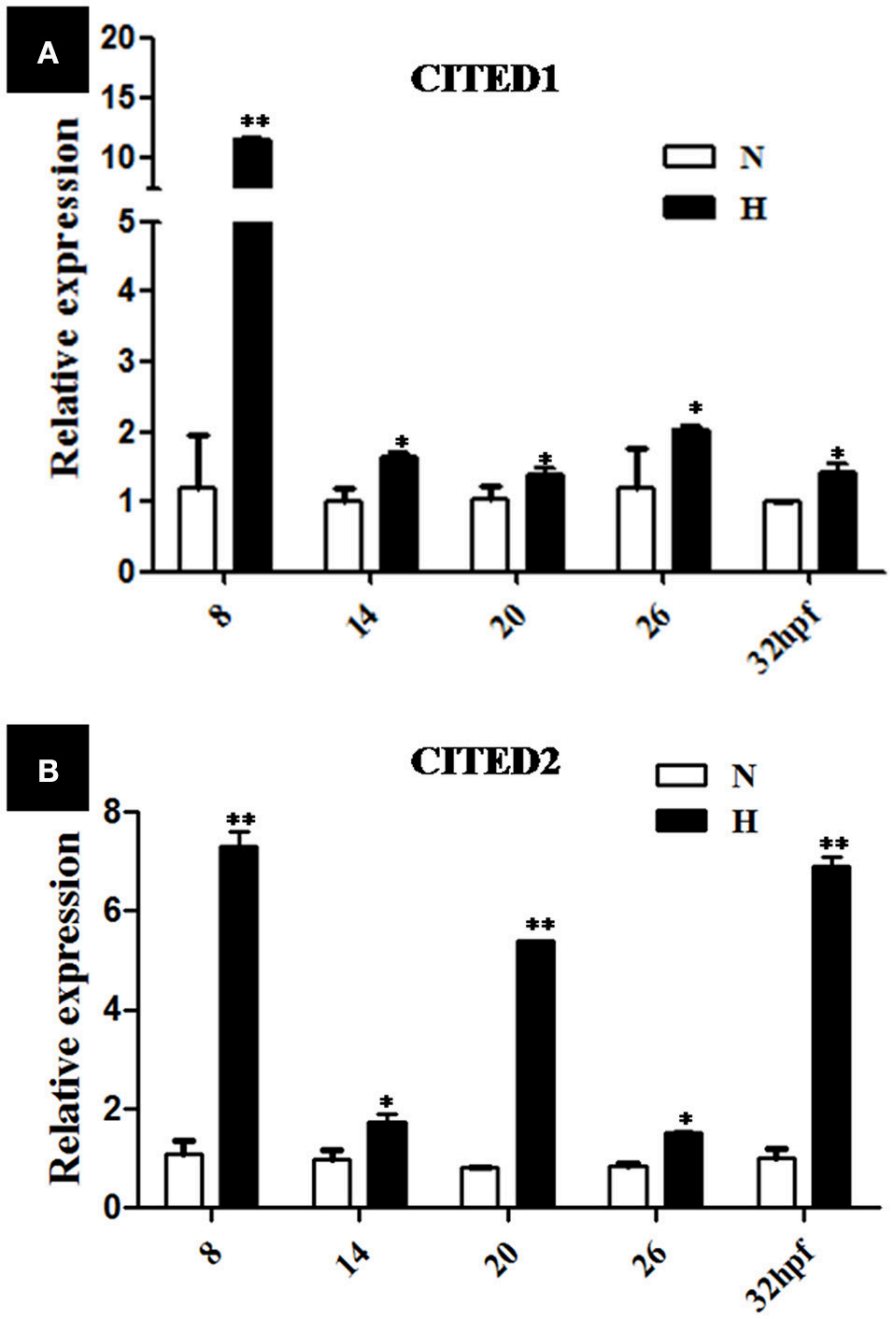
*q*RT-PCR analysis of *CITED1* **(A)** and *-2* **(B)** mRNAs at different development stages of blunt snout bream embryos after treatment with hypoxia (DO = 3.5 ± 0.5 mg/L) for 6 h starting at 2-, 8-, 14-, 20-, or 26 hpf stage. H, hypoxia treatment (DO = 3.5 ± 0.5 mg/L) for 6 h starting; N, normoxia (DO = 7.0 ± 0.5 mg/L), The results are presented as mean ± SE (*n* = 5), ^*^p < 0.01; ^**^p < 0.001.

Various stages of the embryos (2, 8, 14, 20, and 26 hpf) were subjected to hypoxic treatment (DO = 3.5 ± 0.5 mg/L) for 6 h. Embryos from the normoxia group served as controls for every time point. As indicated by *q*PCR analysis, the relative transcriptional level of *CITED1* and *CITED2* significantly (*p* < 0.01) increased after 6 h hypoxia treatment at different stages as compared with the controls (Figures 5A,B). In particular, the relative transcriptional level of *CITED1* at 8 hpf and *CITED2* at 8, 20, and 32 hpf was 10, 7, 5, and 7 times higher, respectively than that observed in the controls at the same developmental stage after 6 h hypoxia treatment (Figures 5A,B).

**Figure 6:**
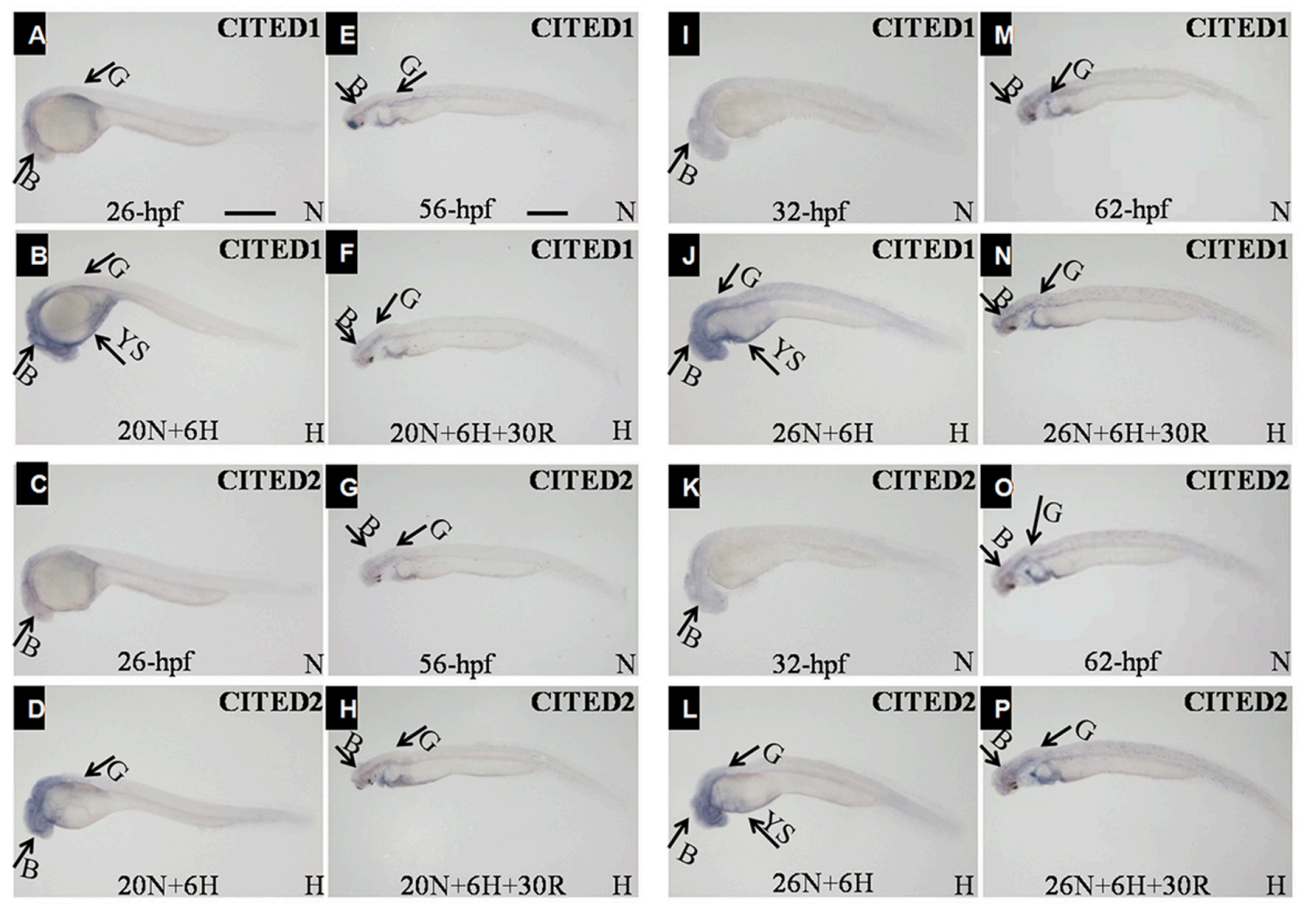
Whole-mount embryo *in situ* hybridization analysis using the *CITED1* **(A,B,E,F,I,J,M,N)** or *-2* **(C,D,G,H,K,L,O,P)** antisense probe. All embryos are viewed laterally with the head to the left. B, brain; G, gut; TB, tail bud; YS, yolk sac; H, hypoxia (DO = 3.5 ± 0.5 mg/L); N, normoxia (DO = 7.0 ± 0.5 mg/L); Scale bar = 500 μm. The embryos at **(B**,**D**) experienced hypoxia treatment for 6 h starting at 20 hpf. The embryos at **(F,H)** embryos are 30 h recovery for the embryos at **(B,D)**. The embryos at **(J,L)** experienced hypoxia treatment for 6 h starting at 26 hpf. The embryos at **(N,P)** embryos are 30 h recovery for the embryos at **(J,L)**.

In comparison with the control embryos at 26 hpf (Figures 6A,C), those under hypoxia showed an obvious increase in levels of both *CITED1* and *CITED2* in the brain. In addition, the transcriptional regions of both genes in the brain were expanded under hypoxia (Figures 6B,D). Additional *CITED1* signals were detected in the yolk sac and trunk mesoderm tissue of hypoxia-treated embryos (Figure 6B). Moreover, mRNA levels of both *CITED1* and *CITED2* at 56 hpf embryos after 30 h recovery (Figures 6F,H) were similar to those observed for controls from same development stage (Figures 6E,G). An increase in the mRNA expression of *CITED1* and *CITED2* was observed in 32 hpf embryos after 6 h hypoxia treatment (Figures 6J,L) as compared with the control at the same stage (Figures 6I,K); the expression level was restored in 62 hpf embryos after 30 h recovery (Figures 6M–P).

### Transcriptional responses of *CITED1* and *CITED2* in juvenile fish under hypoxia stress

**Figure 7:**
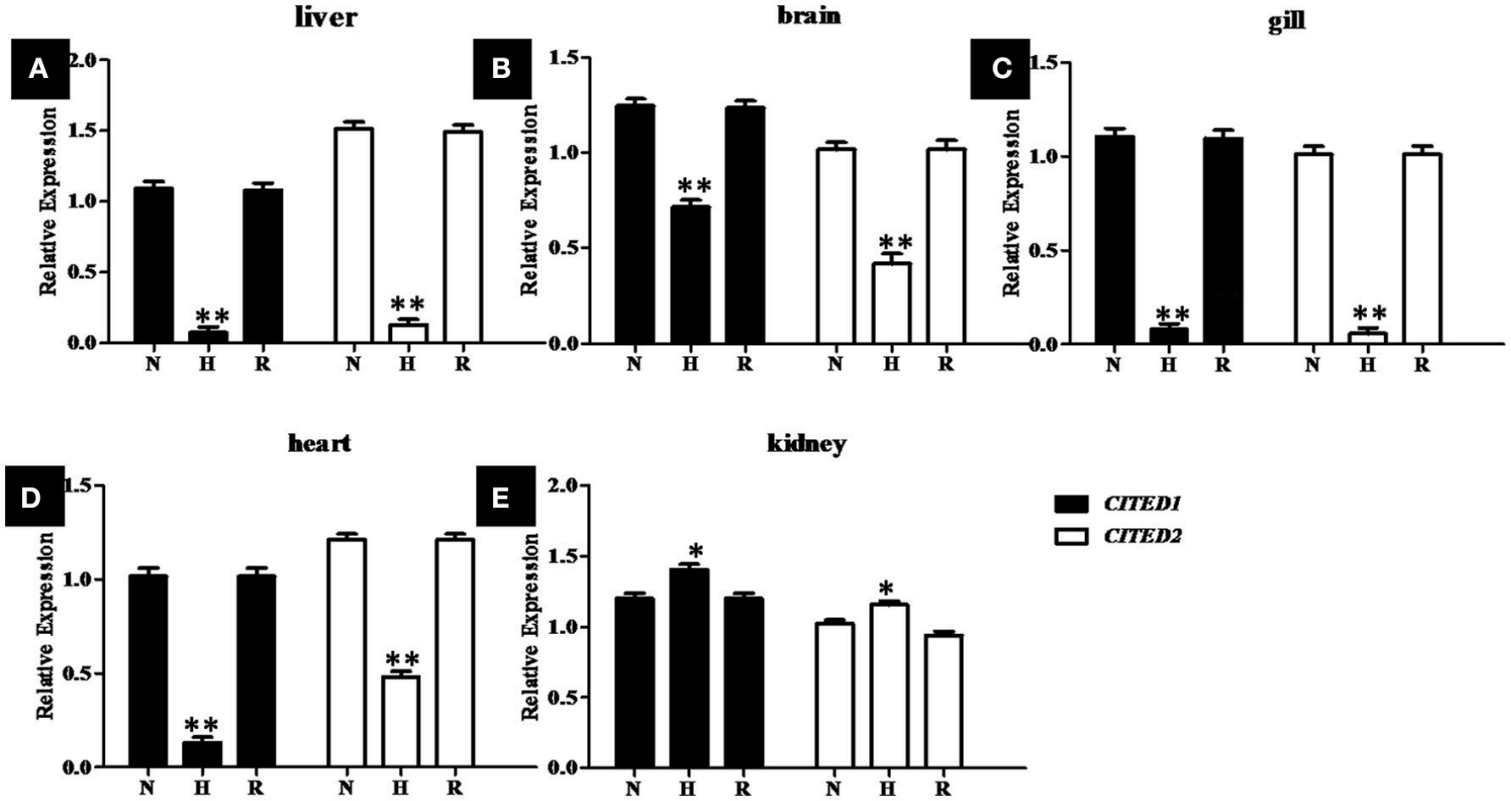
Relative levels of CITED1 and *-2* transcripts in liver **(A)**, brain **(B)**, gill **(C)**, heart **(D)**, and kidney **(E)** during normoxic (N), hypoxic (H) and recovery (R) conditions of blunt snout bream. H: 4 h hypoxia (DO = 1.0 ± 0.5 mg/L) treatment; R, 24 h normoxic recovery (DO = 7.0 ± 0.5 mg/L); N, DO = 7.0 ± 0.5 mg/L. The *CITED1*/−2 copy numbers were normalized to the amount of 18S mRNA. qRT-PCR data presented as mean ± SE (n = 5); ^*^p < 0.01; ^**^p < 0.001.

To determine whether blunt snout bream *CITED1* and *CITED2* genes were involved in the transcriptional response to hypoxia, juvenile fish were exposed to normoxia (DO = 7.0 ± 0.5 mg/L) and hypoxia (DO = 1.0 ± 0.5 mg/L) conditions. The relative expression of *CITED1* and *CITED2* mRNAs in the liver, brain, gill, heart, and kidney during hypoxic treatment were estimated by qPCR (Figure 7). The experimental data suggest that *CITED1* and *CITED2* shared a similar responsive mode at the transcriptional level. Although *CITED1* and *CITED2* mRNA were significantly (*p* < 0.01) upregulated in the kidney (Figure 7E), a significant (*p* < 0.001) downregulation in the expression of these mRNAs was observed in the liver, brain, gill, and heart (Figures 7A–D) during hypoxic stress. After a recovery stage of 24 h, both *CITED1* and *CITED2* mRNA expressions returned to the normal level in various tissues (Figure 7).

## Discussion

In this study, we isolated and characterized two *CITED1* and *CITED2* genes in blunt snout bream, a commercially hypoxia-sensitive aquaculture fish species in China. Blunt snout bream CITED1 and CITED2 mature peptides are very similar to other teleost CITED1 and CITED2 peptides (~90% identity). However, the overall sequence identity between CITED1 and CITED2 mature peptides was only 45%. These observations are in line with those previously reported in grass carp (Ng et al., 2003). Each *CITED1* and *CITED2* gene clustered with its mammalian homologous gene, suggesting that these are not derived from fish-specific genome duplication (Taylor et al., 2003).

Both CITED1 and CITED2 mature peptides of blunt snout bream comprised two highly conserved CR1 and CR2 domains with a highly conserved signature motif. Domain CR3 is absent in CITED1 peptide (Dunwoodie et al., 1998; Bhattacharya et al., 1999). CR2 domain displaying a leucine-rich nuclear-export signal is the characteristic domain of the CITED protein family. It is known that CR2 domain is crucial, especially during the function of CITED1 and CITED2 as transcription activators (Shioda et al., 1997). Through CR2 domain, CITED 1 and CITED2 compete with HIF-1 for binding to CH1 domain of CBP/p300 (Bhattacharya et al., 1999; Fox et al., 2004; Ng et al., 2010). The conservation CR2 domain may imply structural and functional similarities between CITED proteins during evolution.

During embryogenesis, both *CITED1* and *CITED2* mRNAs of blunt snout bream showed fluctuated expression from the zygote to 44 hpf larval stage. They were mainly transcribed in the brain at different embryonic development stages. Our results were consistent with the observation reported in zebrafish, wherein strong *CITED2* transcriptional signals were detected in the brain and some parts of cephalic nervous system at similar embryonic stages (Thisse et al., 2004). Moreover, gene knockout studies have shown that *CITED2* is required for normal embryonic development. Neural crest defects, exencephaly and other developmental defects were found in mice lacking *CITED2* (Bamforth et al., 2001). Furthermore, the expression of *CITED1* were detected at the brain in adult and developing murine (Gerstner and Landry, 2007). Despite the lack of transcriptional or translational information on *CITED1* and *CITED2* in spatial embryo of other fish species, our results suggest that duplicated *CITED1* and *CITED2* genes in blunt snout bream may play overlapping biological roles in the development of cephalic nervous system at transcriptional level.

Oxygen is undoubtedly of significant importance for life but may be influenced by changing circumstances. Cells of fish and other animals can maintain oxygen homeostasis as a resistance mechanism to hypoxic stress by modulating the expression level of related genes (Yachie et al., 1999; Semenza, 2001; Giaccia et al., 2004; Nilsson and Ostlund-Nilsson, 2004). Studies have shown that the transcription of a series of related genes may be upregulated or downregulated during the process of adapting hypoxia condition (Gracey et al., 2001; Terova et al., 2008; Zhang et al., 2017). The recurrence of even short-term hypoxia can affect the hypoxia response (Rytkönen et al., 2012). During embryogenesis, mRNA levels of *CITED1* and *CITED2* were significantly increased in blunt snout bream during various stages (2, 8, 14, 20, and 26 hpf) under hypoxia treatment. Moreover, mRNA levels of both *CITED1* and *CITED2* were obviously enhanced in the brain and their transcriptional areas are expanded. The transcriptional signal returned to normal levels after 30 h of recovery. Although no data are available on embryonic hypoxia regulation in other fish species, our results demonstrated that blunt snout bream *CITED1* and *CITED2* mRNAs are inducible in response to hypoxia during embryogenesis.

Blunt snout bream *CITED1* and *CITED2* mRNA are ubiquitously transcribed in numerous tissues, as observed in mammals and other teleost fish species (Dunwoodie et al., 1998; Gerstner and Landry, 2007; Ng et al., 2010). The highest transcription of *CITED1* mRNA were detected in the gill, spleen, and gonad of blunt snout bream. However, the highest transcription was detected in heart and brain in case of grass carp (Ng et al., 2010). The difference in the expression pattern of *CITED1* gene suggests that this gene may have an adaptive expression pattern in different fish species. The lowest transcription of *CITED1* and *CITED2* mRNA was observed in the muscle of blunt snout bream, similar to that reported in the previous study on *CITED1* in grass carp (Ng et al., 2009, 2010).

After 4 h hypoxia treatment, both *CITED1* and *CITED2* mRNAs levels were significantly downregulated in most of tissues except kidney. This downregulation may account for the negative feedback loop for HIF-1 transactivation, wherein these compete for the transcriptional co-activator CBP/p300 (van den Beucken et al., 2007). Under hypoxia conditions, the decrease in the transcription of *CITED1* and *CITED2* may weaken their capacities to combine with CBP/p300. This contributes to the formation of CBP/p300-HIF complexes that promote the translation of the related downstream genes (van den Beucken et al., 2007). *CITED1* mRNA was significantly upregulated in kidney, but its expression was markedly increased in the heart and liver in grass carp (Ng et al., 2003, 2009, 2010). These differential regulations in the transcription of *CITED1* and *CITED2* may contribute to differences in the tolerance to hypoxia between blunt snout bream and grass carp, although further studies are needed to verify this hypothesis.

In conclusion, we successfully characterized the two distinct *CITED1* and *CITED2* genes in blunt snout bream. Their molecular and spatiotemporal transcriptional patterns and their responses to acute hypoxia suggest that these genes have divergently evolved and may play overlapping biological roles in regulation of response to hypoxia in blunt snout bream. Hypoxic treatment may upregulate *CITED1* and *CITED2* transcription in embryos, but decrease their expressions in multiple adult tissues. Our results may provide new insights into the divergence of *CITED1* and *CITED2* genes and their possible transcriptional or translational regulation in response to hypoxia.

## Author contributions

S-MZ and X-YJ designed experiments; YS and H-HG carried out experiments; YS analyzed experimental results. YS and D-DG analyzed sequencing data and developed analysis tools. YS and S-MZ wrote the manuscript.

### Conflict of interest statement

The authors declare that the research was conducted in the absence of any commercial or financial relationships that could be construed as a potential conflict of interest.
